# Organ Biodistribution of Radiolabelled γδ T Cells Following Liposomal Alendronate Administration in Different Mouse Tumour Models

**DOI:** 10.7150/ntno.32876

**Published:** 2020-02-06

**Authors:** Julie T-W. Wang, Naomi O. Hodgins, Wafa' T. Al-Jamal, John Maher, Jane K. Sosabowski, Khuloud T. Al-Jamal

**Affiliations:** 1 School of Cancer and Pharmaceutical Sciences, King's College London, Franklin-Wilkins Building, 150 Stamford Street, London SE1 9NH, United Kingdom; 2 School of Pharmacy, Queen's University Belfast, 97 Lisburn Road, Belfast, BT9 7BL, United Kingdom; 3 Barts Cancer Institute, Queen Mary University of London, London EC1M 6BQ, United Kingdom

**Keywords:** bisphosphonates, γδ T cells, liposomes, adoptive immunotherapy, nitrogen-containing bisphosphonates

## Abstract

Vγ9Vδ2 T cell immunotherapy has been shown to be effective in delaying tumour growth in both pre-clinical and clinical studies. It has been pointed out the importance of the ability of cells to accumulate within tumours and the association with therapeutic efficacy in clinical studies of adoptive T cell transfer. We have previously reported that alendronate liposomes (L-ALD) increase the efficacy of this therapy after localised or systemic injection of γδ T cells in mice, inoculated with ovarian, melanoma, pancreatic or experimental lung metastasis tumour models, respectively. This study aimed to examine the organ biodistribution and tumour uptake of human γδ T cells in subcutaneous (SC), intraperitoneal (IP) or experimental metastatic lung tumours, established in NOD-SCID gamma (NSG) mice using the melanoma cell line A375Pβ6.luc. pre-injected with L-ALD. Overall, small variations in blood profiles and organ biodistribution of γδ T cells among the different tumour models were observed. Exceptionally, IP-tumour and experimental metastatic lung-tumour bearing mice pre-injected with L-ALD showed a significant decrease in liver accumulation, and highest uptake of γδ T cells in lungs and tumour-bearing lungs, respectively. Lower γδ T cell count was found in the SC and IP tumours.

## Introduction

Vγ9Vδ2 T cells have been shown to be effective as a cell based cancer immunotherapy 1. A major limiting factor to the therapeutic efficacy of cell-based immunotherapy is the ability of the immunologically- functional T cells to accumulate in tumours 2-4. In clinical studies of adoptive T cell transfer, it was found that only a small percentage of the cells reach the tumour tissue 2, 3, and that T cells localisation to tumour correlates well with positive clinical outcomes 3.

Nitrogen-containing bisphosphonates (N-BPs) further increase the efficacy of this immunotherapy in both pre-clinical 5-12 and clinical studies 13-15 as they lead to the accumulation of phosphorylated metabolites that act as natural phospho-antigens for Vγ9Vδ2 T cells 16. Di Carlo *et al*. demonstrated that infiltration of γδ T cells was significantly higher in tumours from mice pre-treated with the N-BP, zoledronic acid (ZOL), than when injected with γδ T cells alone 7. Similarly, in a study by Santolaria *et al.,* immunohistochemical analysis showed the presence of infiltrating γδ T cells only within tumours of NSG mice that received both the N-BP, pamidronate and γδ T cells 10. In addition to the need for Vγ9Vδ2 T cells to infiltrate the tumours, combining the treatment with N-BPs seems to be crucial to achieving positive therapeutic outcome in patients.

Due to the pharmacokinetic properties of N-BPs 17, their encapsulation in liposomes can increase levels of N-BPs in solid tumours 18, 19. Using an ovarian tumour model established by intraperitoneal (IP) inoculation, liposomal alendronate (L-ALD) has been shown to be more effective at slowing tumour growth than ALD when administered intravenously in combination with Vγ9Vδ2 T cells that were injected into the peritoneal cavity of mice 9. Additionally, we have recently reported that only the combinatory treatment of L-ALD and γδ T cells led to a significant reduction in tumour growth in the experimental metastatic lung melanoma model, after 3 successive intravenous injections 20, 21.

Uptake of human γδ T cells in mice has been mostly examined qualitatively in tumours and other organs such as lymph nodes and spleen by immunohistochemical analysis 7, 10, 22, 23. Quantitative assessments on whole body and tumour biodistribution of γδ T cells have been studied in syngeneic 24 or xenograft 23 tumour models injecting murine or human γδ T cells, respectively. This work aims to quantitatively compare, and for the first time, the biodistribution profiles of human γδ T cells in immune-compromised mice, implanted with human melanoma A375 Pβ6 tumours at three different locations: subcutaneous (SC), intraperitoneal (IP) or experimental metastatic lung tumours. Tumour-bearing mice were pre-injected with free form of ALD or L-ALD, followed by infusion of γδ T cells. We investigated whether the different immunogenicity and tumour microenvironment due to the site of tumour implantation will impact the γδ T cell biodistribution and localisation to tumours.

## Methods

### Materials

1,2-distearoyl-*sn*-glycero-3-phosphoethanolamine-N-diethylenetriaminepentaacetic acid (ammonium salt) (DSPE-DTPA) was purchased from Avanti Polar Lipids, Inc (USA). 1,2-distearoyl-sn-glycero-3-phosphocholine (DSPC) and 1,2-dipalmitoyl 1,2-distearoyl-sn-glycero-3-phosphoethanolamine-N-[methoxy(polyethylene glycol)-2000] (ammonium salt) (DSPE-PEG2000) were obtained from Lipoid (Germany). Dextrose, cholesterol, sodium chloride, phosphate buffered saline (PBS) tablet, N-(2-Hydroxyethyl)piperazine-N′-(2-ethanesulfonic acid) (HEPES), methanol (Analytical reagent grade), chloroform (analytical reagent grade), human AB serum (male), ferric chloride hexahydrate, ammonium thiocyanate and copper (II) sulphate pentahydrate were purchased from Sigma-Aldrich (UK). Snake Skin® dialysis tubing (MWCO 10000 Da) was purchased from Thermo-fisher (USA). Dulbecco's modified Eagle's medium (DMEM), Roswell Park Memorial Institute medium (RPMI), GlutaMAX™ and antibiotic-antimycotic solution were purchased from Invitrogen (UK). Foetal Bovine Serum was purchased from First Link (UK). Ficoll-Paque Plus was purchased from GE Healthcare (UK). Citrate-dextrose Solution was purchased from SLS (UK). IL-2 (100U/ml) (Proleukin®) was obtained from Prometheus (USA). T Cell Receptor (TCR) Pan gamma/delta-FITC and IgG FITC Isotype controls were purchased from Beckman Coulter (UK). Tropolone and alendronate sodium trihydrate were obtained from Alfa Aesar (UK). D-Luciferin was obtained from Perkin Elmer (UK). PD-10 and NAP-5 desalting column was obtained from GE Healthcare Life Sciences (UK). Indium-111 chloride (^111^InCl_3_) was obtained from Mallinckrodt (NL). Thin layer chromatography (TLC) strips for radio-labelling were purchased from Agilent Technologies UK Ltd** (**UK). Isoflurane (IsoFlo®) for anaesthesia was purchased from Abbott Laboratories Ltd (UK). All reagents were used without further purification.

### Preparation of liposomes

Lipid stock solutions were prepared in chloroform/methanol (4:1 v/v) at concentrations of 20-40 mg/ml and were stored under nitrogen at -20°C to avoid degradation. Alendronic acid liposomes (L-ALD) were prepared by the thin film hydration (TFH) method as previously described 20, 21, 25. DSPC, cholesterol and DSPE-PEG2000 (55:40:5 molar volume) were added to a 25 ml round-bottom flask and 2 ml chloroform/ methanol (4:1 v/v) was added. A thin lipid film was formed upon removal of the solvent under reduced pressure using a rotary evaporator (Rotavapor® R-210, Buchi UK). The lipid film was flushed with nitrogen to remove any remaining traces of organic solvent. The film was then hydrated with 1 ml of 100 mM ALD in HEPES Buffered Saline (HBS; 20 mM HEPES and 150 mM NaCl), adjusted to pH 7.4. The liposome suspension was left for 1 h at 60 °C and was vortexed (Vortex genie 2, Scientific Industries Inc, USA) every 15 min 25. The resulting suspension was stored at 4°C. Liposome's size and polydispersity (PDI) were reduced with serial extrusion using the mini-extruder (Avanti Polar Lipids, USA) through polycarbonate membranes (Avanti Polar Lipids, USA) with pore sizes 0.8 µm (5x), 0.2 µm (5x), 0.1 µm (10x) and 0.08 µm (15x), at 60°C. Free ALD was separated from the liposomes by dialysis against HBS using a 10 kD dialysis bag. L-ALD were prepared at a final lipid concentration of 20 mM.

### Physicochemical characterisation of liposomes

The hydrodynamic diameter, polydispersity index and zeta potential of the liposomes were measured using the NanoZS (Malvern Instrument, UK). Hydrodynamic size, polydispersity index (PDI) and zeta potential were measured in disposable square polystyrene cuvettes and disposable capillary cells, respectively (Malvern Instrument, UK). The sample (20 µl) was diluted to 1.5 ml with 10 mM sodium chloride. The measurements were carried out at room temperature. Three measurements were performed, and the mean and standard deviation were calculated for each sample.

### Quantification of ALD

ALD concentrations, for determination of % EE, were determined with a copper sulphate-based UV detection method 26. A calibration curve containing 5 mM empty liposomes and between 0.1-1 mM of free ALD, referred to 'ALD spiked liposomes' samples, was prepared. L-ALD samples to be quantified (or standards) were both processed using the Folch method prior to quantification. This method disrupts the liposomes; allowing the encapsulated ALD to be released into the aqueous phase and separating any hydrophobic components (cholesterol, lipids etc.) from the hydrophilic drug. In brief, chloroform and methanol were added to a sample of liposomes at 8:4:3 (chloroform: methanol: liposomes) volume ratio. The sample was then vortexed (Vortex genie 2, Scientific Industries Inc, USA) and centrifuged at 10000 rpm for 10 minutes (Centrifuge 5810 R, Eppendorf). Two layers were formed after centrifugation and the upper aqueous layer containing the ALD was removed and quantified. CuSO_4_ reagent was prepared by dissolving 10 mM CuSO_4_ in deionised water. A 0.5 ml sample of the upper aqueous phase was to 0.5 ml of 10 mM CuSO_4_ reagent. After 10 minutes, the UV absorbance at 240 nm was measured with a UV/Vis Spectrophotometer (Perkin Elmer, Model: Lambda 35), using HBS as the reagent blank. Concentrations were calculated from the equation obtained from the 'ALD spiked liposomes' calibration curve. Encapsulation Efficiency (EE %) was calculated was calculated by comparing the ALD concentration of liposomes to the concentration before purification. The quantity of N-BP in each liposome sample was measured three times and expressed as mean ± standard.

### Isolation and expansion of Vγ9Vδ2 T cells

The γδ T cells were isolated and expanded as previously described 20, 21. Blood samples of 20-30 ml were obtained from healthy volunteers in an ethically approved protocol (“Use of Donor Blood Samples for Pre-Clinical Development of Active and Passive Immunotherapy for Cancer”; Ref.09/H0804/92). The blood sample was added to 5 ml of citrate-dextrose solution to prevent clotting. The sample was then layered on top of 15 ml of Ficoll-Paque Plus and centrifuged at 1150 g for 25 min, with no acceleration or break, using a bench centrifuge (Centrifuge 5810 R, Eppendorf). The layer of cells between the Ficoll-Paque Plus and the plasma was then removed and the resulting PBMCs were washed twice with PBS and were then suspended in RPMI 1640 (containing 10% human AB serum, 1% Glutamax and 1% antibiotic-antimycotic solution) at a concentration of 3x10^6^ cells/ml. In order to expand the Vγ9Vδ2 T cells, the PBMCs were activated with 1 µg/ml ZOL and 100 U/ml IL-2. Additional medium and 100 U/ml IL-2 were added every 2-3 days for 15 days.

### Flow cytometry analysis of γδ T cells

On Day 1 and Day 15, 200 µl samples of the cell suspension were taken and 5 µl of either T Cell Receptor (TCR) Pan γ/δ-FITC antibody or IgG1 FITC Isotypic control antibody was added. The cells were incubated with the antibodies for 20 min at 4°C before 1 ml PBS was added. Cells were centrifuged at 1000 rpm for 5 min in a bench centrifuge (Centrifuge 5810 R, Eppendorf). The supernatant was discarded and the cell pellet was re-suspended in 500 µL of PBS. All flow cytometric data were acquired using a Beckman Coulter Cytometer FC 500 MPL and were analysed using CXP Analysis software (Beckmann Coulter). The lymphocyte cell population was gated and the number of cells in this gate that express the γδ TCR were calculated as a percentage of the total lymphocytes.

### Cancer cell line culture conditions

The cell line A375Pβ6 was created using the human melanoma cell line A375P (CRL-3224™), which was infected with pBabe retroviruses encoding puromycin resistance and cDNA for human β6 integrin (A375Pβ6), as previously reported 27. This cell line was a kind gift from Prof John Marshall (QMUL). The A375Pβ6 cell line was subsequently transduced as described 9 with an SFG retroviral vector that encodes for firefly luciferase (luc) and dsTomato red fluorescent protein to allow tumour growth monitoring. Transduced cells were then flow sorted for red fluorescent to obtain a pure A375Pβ6.luc cell line. The cell lines were maintained at 37°C, 5% CO_2_ and 5% relative humidity. DMEM media was used, supplemented with 10% FBS, 1% GlutaMAX™ and 1% Penicillin/Streptomycin.

### Radiolabelling of liposomes

Liposomes containing alendronate (L-ALD) were prepared and characterised for their size, charge and drug loading as previously described 20, 21. Compositions are DSPC, cholesterol, DSPE-PEG2000 and DSPE-DTPA (54:40:5:1 molar volume). Liposomes were then radiolabelled with ^111^In using protocols as described previously 20, 21. In brief, the required volume of ^111^In, containing 1 MBq per mouse for gamma counting studies, was added to 2 M ammonium acetate buffer (one-ninth of the reaction volume, pH 5.5). This was then added to the liposome sample (100 μl of 20 mM liposomes/mouse) to give a final ammonium acetate concentration of 0.2 M. The mixture was incubated for 30 min at room temperature with vortexing every 10 min. The reaction was quenched by the addition of 0.1 M EDTA solution to the mixture (5 % v/v of the reaction mixture) to chelate free [^111^In]. Unbound [^111^In]EDTA was removed using NAP-5 desalting columns equilibrated with PBS with the liposomes collected in fractions 1-3 (~150 μl per injection dose). Labelling efficiency was assessed as previously reported 20.

### Efficiency and stability of the radiolabelled liposomes

Samples of the radiolabelled L-ALD or [^111^In]EDTA were spotted in glass microfibre chromatography paper impregnated with silica gel. These strips were then developed using a mobile phase of 50 mM EDTA in 0.1 M ammonium acetate. Strips were placed on a multi-purpose storage phosphor screen (Cyclone ®, Packard, Japan) and kept in an autoradiography cassette (Kodak Biomax Cassette ®) for 10 min. Quantitative autoradiography counting was then carried out using a cyclone phosphor detector (Packard ®, Australia). The labelling stability was tested by incubation of the radio-conjugates in the presence or absence of foetal bovine serum (FBS). Samples were diluted in 50% FBS or PBS [1:2 (v/v)], and incubated for 24 h at 37°C. The percentage of [^111^In] (immobile spot) still conjugated to the liposomes was evaluated by TLC, using the same protocol as described above.

### Radiolabelling of γδ T cells

The γδ T cells were isolated and expanded as previously described 20 (See Supplementary Information). The percentage of γδ T cells obtained from healthy volunteers were variable from batch to batch, ranging from 0.8 to 28.3 % of the total lymphocytes, assessed by flow cytometry. After isolation and expansion in culture, γδ T cell population generally increased to above 80 % on Day 15. The number of cells to be used for radiolabelling were calculated based on the percentage of γδ T cells harvested. Cell were radiolabelled using [^111^In]tropolone following reported methods with modification 28-30. Tropolone was dissolved in isotonic HEPES buffered saline (HBS) at a concentration of 1 mg/ml to form a stock solution. A volume of ^111^In stock (as ^111^InCl_3_), containing 1 MBq (2 ul/mouse) or 10-15 MBq (20-30 µl/mouse) per mouse for gamma counting or SPECT/CT imaging studies, respectively, was added to a volume of the tropolone stock solution (3.6 or 36-54 ul/mouse) at a ratio of 5:9 v:v (^111^InCl_3_ stock_:_ tropolone stock) allowing for [^111^In]tropolone complex to form. The [^111^In]tropolone mixture was added to the γδ T cells (5 x 10^6^/mouse in 50 µl) and incubated for 20 minutes in an incubator at 37°C, 5% CO_2_ and 5% relative humidity with gentle mixing of the cells every 5 min. Any [^111^In]tropolone not incorporated into the cells was removed by centrifuging the cells for 10 min at 500 g and discarding the supernatant. The cells were washed twice with 10 ml PBS and were then finally re-suspended in PBS at a concentration of 5 x 10^6^ cells per 150 μl.

### Animal models

All animal experiments were performed in compliance with the UK Home Office (1989) Code of Practice for the housing and care of Animals used in Scientific Procedures. SCID/Beige mice (used in SPECT/CT studies) and Male NOD SCID gamma (NSG) mice (~20 g), 4-6 weeks old (used in all other studies), were obtained from Charles River (UK). Subcutaneous (SC) tumours were established by injecting 5 x 10^6^ cells A375Pβ6.luc in 100 μl PBS subcutaneously into each of the rear-flanks of the mouse. The size of the tumour was measured using digital callipers and tumour was then determined using the equation:

Tumour Volume (mm^3^) = (*A*^2^*B*π)/6

where *A* and *B* represent the width and the length of the tumours, respectively 31. Experimental metastatic lung tumours were established by slowly injecting 5 x 10^5^ cells in 100 μl PBS i.v. into the tail vein. Intraperitoneal (IP) tumours were established by injecting 5 x 10^6^ cells in 100 μl PBS into the intraperitoneal cavity. Both of these deep tumour models were monitored by detecting the bioluminescence emitted from the A375Pβ6.luc cells 12 min after subcutaneous injection of D-luciferin at 150 mg/kg using an IVIS Lumina series III *In Vivo* Imaging system (Perkin-Elmer, USA). Mice were imaged on day 6 post-inoculation and subsequently every 3-4 days. Images were quantitatively analysed by drawing regions of interest around the tissues using Living Image 4.3.1 Service Pack 2 software (Perkin-Elmer, USA). For tumour inoculation, intravenous injection, blood sampling and imaging, mice were anesthetised using isoflurane inhalation anaesthesia.

### Whole body SPECT/CT imaging of radiolabelled γδ T cells in A375Pβ6 tumour bearing mice

Each mouse was injected with 5 x 10^6^ radiolabelled γδ T cells ([^111^In]γδ T cells) or the equivalent amount of radioactivity as [^111^In]tropolone *via* tail vein injection. Mice were imaged with nanoSPECT/CT scanner (BioscanInc., USA) 0-30 min, 4 h and 24 h after i.v. administration using isoflurane as inhalation anaesthesia. For each mouse, a tomography was initially acquired (45 kVp; 1000 ms) to obtain parameters required for the SPECT and CT scanner, including the starting line, finish line and axis of rotation of the acquisition. SPECT scans were obtained using a 4-head scanner with 1.4 mm pinhole collimators using the following settings: number of projections: 24; time per projection: 60 sec and duration of the scan 60 min. CT scans were obtained at the end of each SPECT acquisition using 45 kVp. All data were reconstructed with MEDISO (medical Imaging System) and the combining of the SPECT and CT acquisitions were performed using VivoQuant (Invicro LLC, USA).

### Gamma counting of radiolabelled γδ T cells and L-ALD in A375Pβ6-tumour bearing mice

For L-ALD biodistribution studies, mice were injected with [^111^In]L-ALD (2 μmol lipid) *via* tail vein injection. For the γδ T cell bio-distribution studies, mice were pre-injected with PBS, 0.5 μmol ALD or L-ALD 24 hours prior to injection with 5 x 10^6^ [^111^In]γδ T cells per mouse. After 24 h, mice were sacrificed and the major organs (brain, lung, liver, spleen, kidney, heart, stomach and intestine), muscle, skin, bone (femur), carcass and tumours were collected, weighed and placed in scintillation vials. Additionally, 5 μl blood samples were taken from the tail vein at various time points (30, 60, 240 and 1440 min for γδ T cells and 5, 10, 30, 60, 240 and 1440 min for L-ALD). Urine and faeces were collected, by housing the mice in metabolic cages for 24 h, to determine the excretion profile. Each sample was analysed for [^111^In] specific activity using an automated gamma counter (LKB Wallac 1282 Compugamma, PerkinElmer, UK) together with dilutions of the injected dose with dead time limit below 60%. The gamma rays emitted by the radioisotope were detected, quantified and corrected for physical radioisotope decay by the gamma counter. Radioactivity readings (counts per minute) were plotted as percentage of injected dose per organ (% ID/organ) or percentage of injected dose per gram of tissue (% ID/g). The data were expressed as the mean of quadruplicate samples ± SD. In the γδ T cells biodistribution studies, with 3 tumour models (SC, IP and metastatic lung models) and 3 pre-treatments (PBS, ALD and L-ALD), a total of 36 mice were used. In the [^111^In]L-ALD biodistribution studies, with 3 tumour models and one time point, a total of 12 mice were used.

### Statistics

For all experiments, data were presented as mean ± SD; *n* denotes the number of repeats. For *in vivo* studies, significant differences were examined using one-way ANOVA. The t-value, degrees of freedom and two-tailed significance (*p*-value) were determined. Kruskal-Wallis test followed by Dunn's test was used for multiple groups comparison. * *p* < 0.05, ** p < 0.01 and *** *p* < 0.001

## Results

### L-ALD has comparable tumour uptake in solid and metastatic A375Pβ6.luc tumour models

L-ALD containing 5% DSPE-PEG_2000_ was formulated using the TFH method. To perform biodistribution studies, DTPA-containing liposomes (DTPA-L-ALD) were formulated by incorporation of 1 mol % DSPE-DTPA in the formulation, to allow for chelation of [^111^In]. The physicochemical characteristics, percentage encapsulation efficiency of ALD (% EE), and radiolabelling efficiency are summarised in **Table 1**. A hydrodynamic size of 152.4 - 156.6 nm, PDI of 0.051 - 0.058, zeta potential of -11.1 to -12.5 mV and % EE of 5.9 - 6.2 % were obtained for L-ALD and DTPA-L-ALD (p > 0.05). [^111^In]DTPA-L-ALD was successfully formed with a radiolabelling efficiency of 82.6 ± 4.2 %. Characterisation results are comparable to what have been reported in our previous study 20.

**Figure 1:**
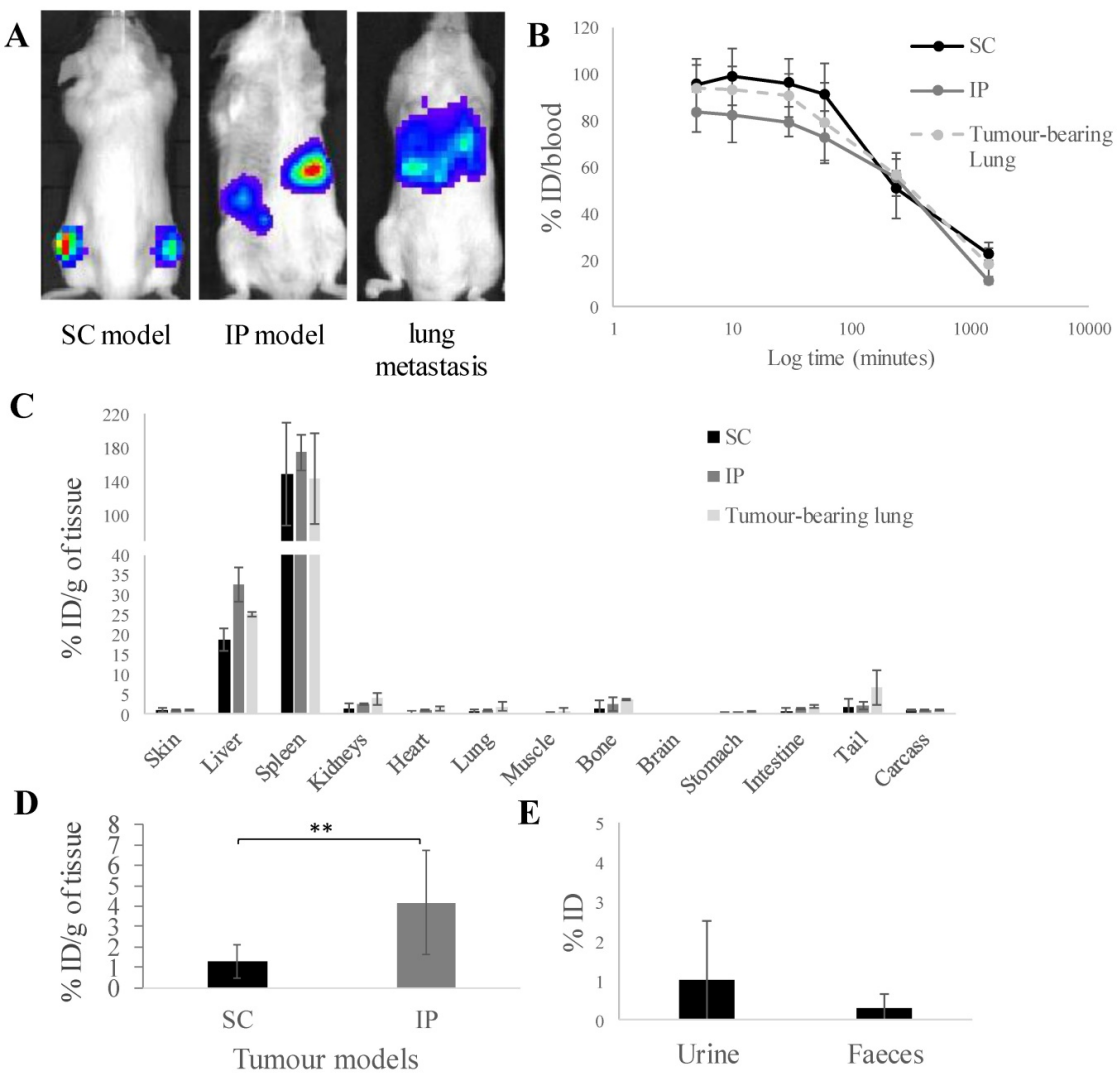
*** In vivo* biodistribution of radiolabelled [^111^In]L-ALD in A375Pβ6.luc tumours, after single dose administration *via* tail vein injection in NSG mice. (A)** Bioluminescence imaging of three A375Pβ6 tumour models in NSG mice. **(B)** Blood clearance profile of [^111^In]L-ALD expressed as % ID. **(C)** Organ biodistribution of [^111^In]L-ALD expressed as per cent injection dose per gram of organ (%ID/g). **(D)** Uptake of [^111^In]L-ALD in SC and IP tumour models at 24 h expressed as %ID/g. **(E)** Excretion profile of [^111^In]L-ALD expressed as % ID. Subcutaneous (SC), intraperitoneal (IP) and pseudo-metastatic lung tumours were established by inoculating NSG mice by i.v. injection of 5 x 10^5^ A375Pβ6 cells, i.p. injection of 5 x 10^5^ A375Pβ6 cells and s.c. injection of 5 x 10^6^ A375Pβ6 cells, respectively. A375Pβ6 cells were transduced with firefly luciferase and tumour growth was monitored by bioluminescence imaging (IVIS Lumina series III *In Vivo* Imaging system, Perkin-Elmer). Tumours were established for three weeks prior to use. Mice were i.v. injected with [^111^In]L-ALD at a dose of 2 μmol lipid/mouse. Blood samples (5 µl) were taken at 5, 10 and 30 min and 1, 4 and 24 h. Urine and faeces were collected. After 24 h the mice were sacrificed, and liposome uptake was quantified by gamma counting. Data was expressed as mean ± SD (n=6-10 mice per group). ** p < 0.01

Three tumour models, subcutaneous (SC), intraperitoneal (IP) and experimental lung metastatic of A375Pβ6.luc tumours were successfully established in NSG mice. Tumour monitoring with representative bioluminescence images are shown in **Figure 1A**. The biodistribution of [^111^In]L-ALD at 24 h after i.v. injection was quantified in tumour bearing NSG mice by gamma counting. This helped us to understand whether there are changes in tissue accumulation of L-ALD, particularly among A375Pβ6.luc tumours inoculated at different locations in mice, which could have an influence on the consequent γδ T cells transfer.

Prolonged blood circulation times were observed with ~55 % ID and ~20 % ID still detected in the blood after 4 h and 24 h, respectively (**Figure 1B**) among the three tumour models. Low amounts of [^111^In]L-ALD was detected in the urine (1.0 ± 0.4 %) and faeces (0.3 ± 0.2 %) (**Figure 1D**) of SC tumour bearing mice. Low amounts of [^111^In]L-ALD excreted to urine and faeces were also observed in the other two tumour models (data not shown). Similar biodistribution profiles in general were found in the three models (**Figure 1C**). As expected, high levels of radioactivity were detected in liver (18.7 - 32.5 % ID/g) and spleen (143.4 - 174.7 % ID/g).

[^111^In]L-ALD uptake in IP tumours was 4.2 ± 2.6 % ID/g, significantly higher than SC tumours (1.3 ± 0.8 % ID/g) (*p* < 0.01). For the lung metastasis model, since the cancer nodules in the lung could not be isolated, [^111^In]L-ALD uptake in the whole lung was assessed. The uptake in tumour-bearing lung (1.2 ± 1.1 % ID/g) was not significantly higher than the lungs of SC model mice (0.7 ± 0.5 %ID/g) and IP model mice (1.0 ± 0.2 %ID/g) (**Figure 1C**). In comparison between the SC and IP tumours, a significantly higher level of [^111^In]L-ALD was detected in IP tumours although large variation in the uptake in the collected tumour nodules was observed (**Figure 1D**). The results of organ biodistribution profiles of [^111^In]L-ALD expressed as %ID/organ is shown in **Figure S1**.

### *In vivo* whole body SPECT/CT imaging of γδ T cells in SC-tumour bearing mice

γδ T cells were labelled with [^111^In]tropolone, producing [^111^In]γδ T cells, to enable biodistribution assessments *in vivo*. Tropolone is a safe and cheap ionophore for [^111^In] shuttling across cell membrane. Tropolone chelates radionuclides and form a complex that allows the radionuclide to be transported across the cell membrane and into the cell lumen. This method is well-established to radiolabel platelets and lymphocytes using [^111^In] as the radionuclide for *in vivo* studies 28-30. Upon incubation with cells, [^111^In]Trop gets translocated into the cellular lumen, forming the intermediate [^111^In]Trop-cell. As the interaction between [^111^In] and tropolone is not particularly strong, ^111^In^3+^ from the [^111^In]Trop will then exchange with proteins and nucleic acids inside the cell. Free tropolone molecules leaves the cell lumen and the ^111^In^3+^ is then entrapped within the lumen, thereby resulting in radiolabelled cells. The average radiolabelling efficiency of ~60 % was achieved (data not shown). The labelling conditions are mild, and the radioactivity used was relatively low compared to other studies, in which most of the cells were expected to remain viable 32.

**Figure 2:**
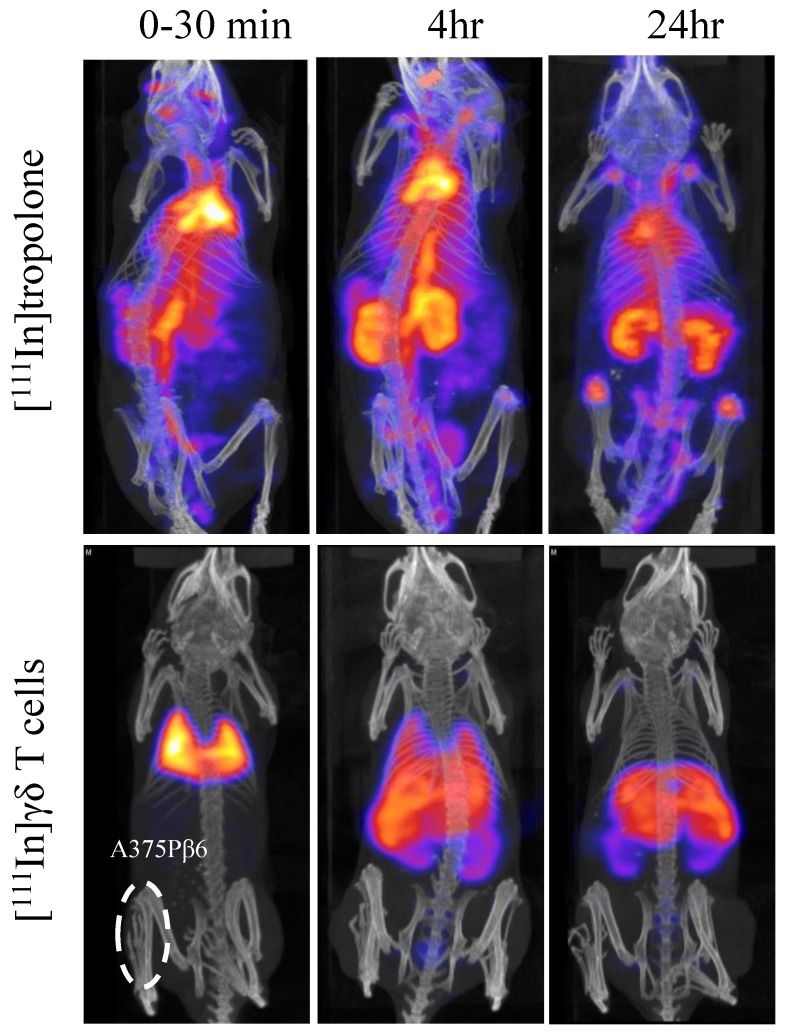
***In vivo* whole body 3D SPECT/CT imaging of [^111^In]tropolone and [^111^In]Vγ9Vδ2 T cells in SC-tumour-bearing SCID/Beige mice.** γδ T cells were radiolabelled by incubation with [^111^In]tropolone. Any [^111^In]tropolone not incorporated into the γδ T cells was removed by centrifugation. Tumour bearing mice were intravenously injected with 5 x 10^6^ [^111^In]γδ T cells or the equivalent amount of [^111^In]tropolone. The mice were imaged immediately and after 4 and 24 h. The γδ T cells go directly to the lung and then redistribute to the liver, spleen and kidneys over time.​ The pattern of biodistribution for [^111^In]tropolone was markedly different to that of the [^111^In]γδ T cells, suggesting that the cells were stably labelled and have been successfully purified from any free [^111^In]tropolone.​

Whole body SPECT/CT imaging of [^111^In]γδ T cells in A375Pβ6 SC-tumour-bearing NSG mice, was performed to track the kinetic changes in organ biodistribution of [^111^In]γδ T cells up to 24 h post-injection (**Figure 2**). [^111^In]γδ T cells were shown to accumulate in the lungs within 30 min post-injection, and redistribute overtime to the liver, spleen, and kidney. SPECT/CT imaging of [^111^In]tropolone in mice was also performed as a control. The pattern of biodistribution for [^111^In]tropolone was markedly different to that of the labelled γδ T cells, showing prolonged circulation and increased kidney excretion overtime. The phenomenon of initial high accumulation of T cells in lung after intravenous injection and the redistribution to liver, spleen and kidney at later time points was in line with the imaging results reported by others after radiolabelling 22, 23. This suggests that the cells were successfully radiolabelled. The signals detected in kidney may correspond to the released [^111^In] from γδ T cells which was also suggested by the other studies 22, 23.

### Altered liver and spleen uptake of γδ T cells was observed in mice pre-treated with L-ALD

SPECT/CT images of γδ T cells in mice bearing SC tumours showed only faint signals in the tumours due to high accumulation of γδ T cells in lung, liver and spleen. Gamma counting, a quantitative method with excellent sensitivity, was consequently performed to assess the biodistribution profiles of γδ T cells at 24 h post-injection in three A375Pβ6.luc tumour models. All mice received pre-treatments of PBS, free ALD or L-ALD intravenously 24 h prior to T cell injection. It is suggested that the subtle alteration in the biodistribution of γδ T cells due to variations in tumour sites or ALD pre-treatments, that cannot be observed by SPECT/CT imaging, can be captured by gamma counting.

**Figure 3:**
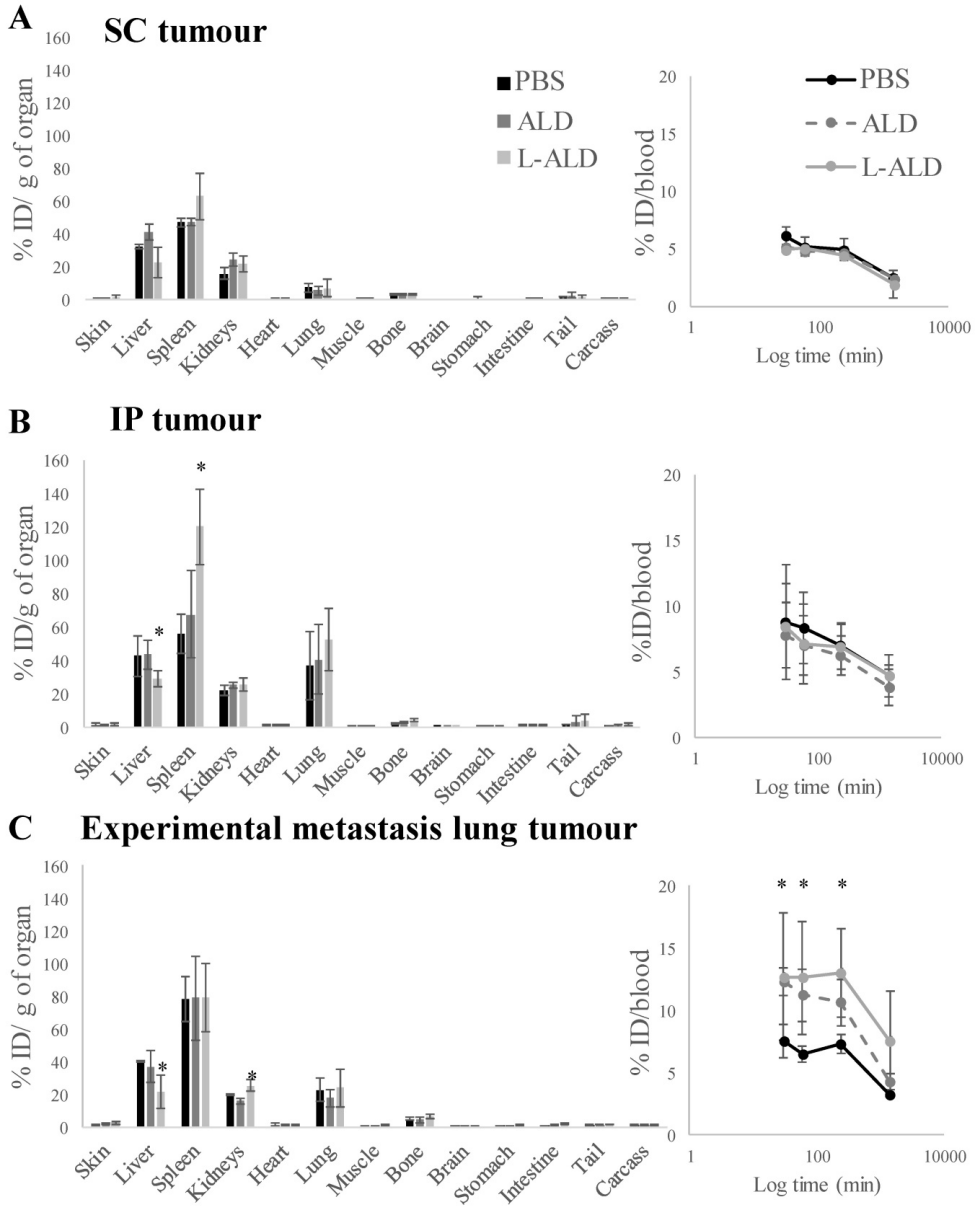
***In vivo* biodistribution of radiolabelled γδ T cells in A375Pβ6.luc tumour bearing NSG mice, after single dose administration *via* tail vein injection.** NSG mice were inoculated with luciferase-expressing A375Pβ6.luc tumour cells to form **(A)** subcutaneous (SC) **(B)** intraperitoneal (IP) or **(C)** experimental metastatic lung tumours. Tumours were allowed to establish for three weeks prior to the biodistribution study. Mice were i.v. injected with [^111^In]γδ T cells at a dose of 5 x 10^6^ γδ T cells/mouse. Mice were pre-treated with 0.5 μmol ALD or L-ALD, 24 h prior to injection of γδ T cells. Blood samples (5 µl) were taken at 0.5, 1, 4 and 24 h. After 24 h the mice were sacrificed and the biodistribution of γδ T cells was quantified by gamma counting. Results are expressed as percent injection dose per gram of organ (%ID/g organ) or % ID/blood for blood samples. Data are expressed as mean ± SD (n=4 mice per group). Statistical significance was indicated in comparison to PBS-injected mice. *p < 0.05, (one-way ANOVA).

In agreement with the SPECT/CT results, the liver, spleen and kidney showed the highest accumulation of [^111^In]γδ T cells. Overall, small variations in blood profiles and organ biodistribution among the different tumour models were observed (**Figure 3 and Figure S2**). Exceptionally, IP-tumour and experimental metastatic lung-tumour bearing mice pre-injected with L-ALD showed a significant decrease in liver accumulation (**Figure 3B and C**, light grey bars, *p* < 0.05), while only IP-tumour bearing mice had an increase in spleen accumulation (**Figure 3B**, light grey bars, *p* < 0.05) compared to PBS-injected mice. Changes in kidney and skin accumulation of γδ T cells were observed on a random basis.

Similar blood profiles of [^111^In]γδ T cells were obtained between different tumour models with or without receiving pre-injection of free form of ALD or L-ALD. An exception was found in experimental lung metastasis model with higher amount of [^111^In]γδ T cells measured in the blood in the mice with pre-injection of free form of ALD or L-ALD up to 4 h post-injection compared to the control ones pre-injected with PBS. Low levels of [^111^In]γδ T cells were present in the urine (less than 0.1 % ID) and the faeces (< 0.5 % ID) in the SC tumour model which could be the result of released [^111^In] as mentioned previously.

### γδ T cells uptake varies among the different tumour locations

**Figure 4:**
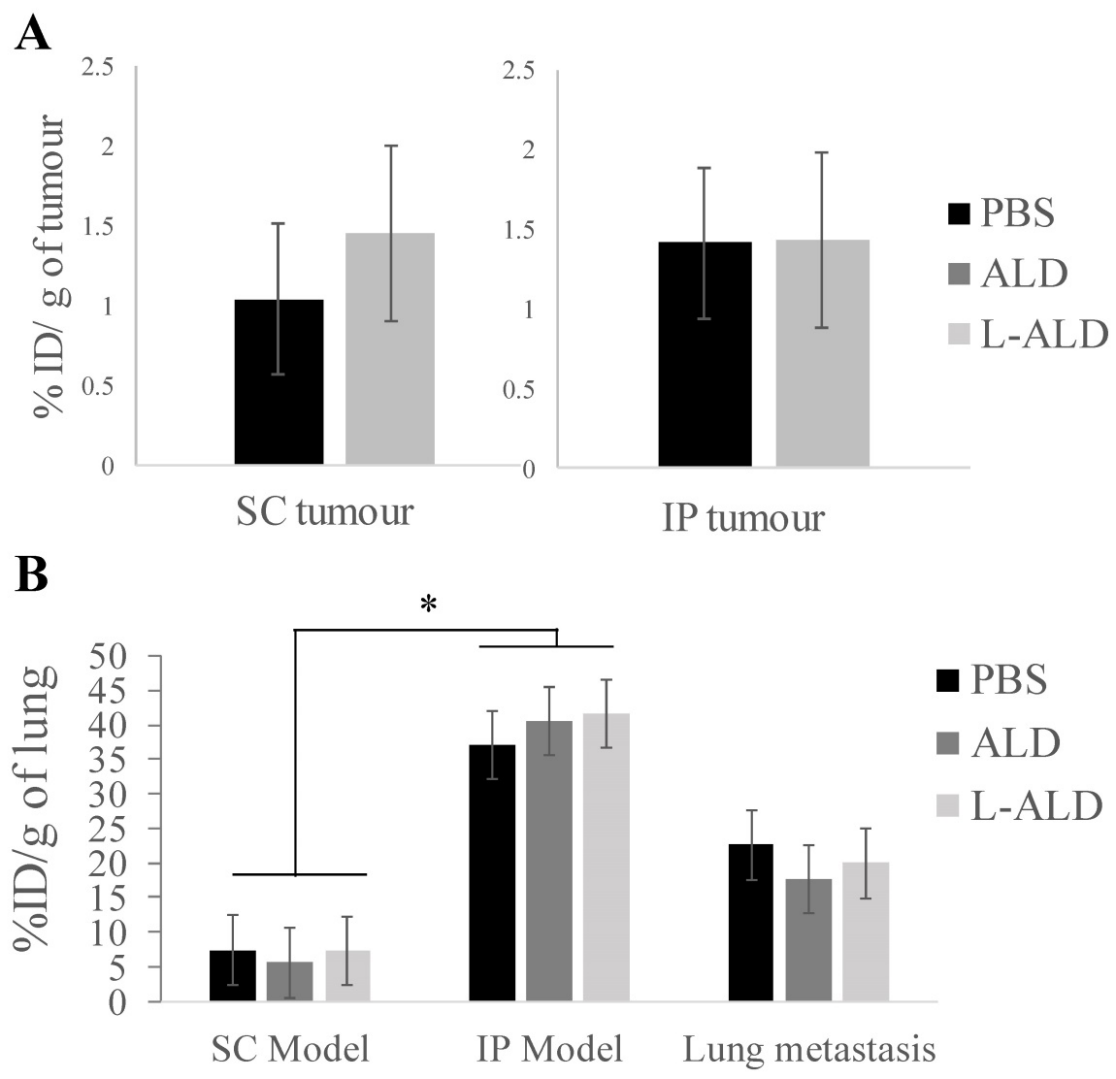
*** In vivo* tumour uptake of radiolabelled [^111^In]γδ T cells in A375Pβ6.luc tumours, after single dose administration *via* tail vein injection in NSG mice.** NSG mice were inoculated with the A375Pβ6.luc cell line to form subcutaneous (SC), intraperitoneal (IP) or experimental metastatic lung tumours. Mice were i.v. injected with 0.5 μmol ALD or L-ALD 24 h prior to i.v. injection of [^111^In]γδ T cells at a dose of 5 x 10^6^ γδ T cells/mouse. **(A)** Tumour uptake of [^111^In]γδ T cells expressed as % ID/g of tumour in SC and IP tumour-bearing mice. **(B)** Lung uptake of [^111^In]γδ T cells expressed as % ID/g of lung in SC, IP and experimental metastatic lung tumour-bearing mice. Pre-injection of ALD or L-ALD did not significantly alter the tumour uptake of γδ T cells. Data are expressed as mean ± SD (n=4 mice per group). Statistical analysis was down by Kruskal-Wallis test followed by Dunn's test to compare the differences between individual group (* *p* < 0.05).

We compared uptake of [^111^In]γδ T cells in tumours in the three tumour models to examine if tumour location influenced their infiltration by γδ T cells. Similar and low uptake of γδ T cells in SC and IP tumours of 1.0 - 1.5 % ID/g tumour was detected **(Figure 4A)**. In the case of the experimental metastatic lung tumour model, the uptake in tumour-bearing lung as a whole was determined and compared to the lung uptake in other models **(Figure 4B)**. Interestingly, the uptake of γδ T cells in the lungs of IP models was significantly higher than that of lungs obtained from SC model mice (*p* < 0.05). The amount of γδ T cells in lung metastasis was not significantly different to the lung uptake either in SC or IP model. Pre-injection of ALD or L-ALD did not change the uptake of γδ T cells in any of the tumours or lungs in the three models studied.

## Discussion

While Vγ9Vδ2 T cells have been shown to be effective as a cancer immunotherapy 1, especially when used in combination with N-BPs 5-15, little is known about the *in vivo* behaviour of human γδ T cells in murine models. This is important to find out if a correlation between the number of γδ T cells in tumour mass and the success of the therapy can be established. While the use of human γδ T cells in cancer therapy is well established, yet questions remain concerning mechanisms involved in recruiting these cells to specific tumours in different tissues. This is an important issue since localisation to the tumour mass is thought to be required for efficacy. Previous studies where human γδ T cells were adoptively transferred in different tumour models established in mice mostly used qualitative, histological approaches to address tumour accumulation 7, 10. In this work a highly quantitative and unbiased approach is used to measure human γδ T cell accumulation in tumours grown in different locations in mice.

Previously, lipophilic chelators such as tropolone and oxine have been used to radiolabel a variety of blood cells for *in vivo* studies 28-30. Many studies have proved that such mild labelling method and condition does not compromise the cell viability. For instance, Jin *et al*. has assessed the impact of labelling with [^111^In]tropolone on cell viability 32. They labelled 5 million primary isolated bone-marrow-derived stem cells with up to 18 MBq [^111^In]tropolone. It was reported that more than 90 % of cells were still viable on day 14 when 5 million cells were exposed to up to 2.5 MBq [^111^In]tropolone. In another study, Tai *et al*. dual-labelled one million pancreatic islet cells with 20 MBq [^111^In]tropolone and implanted the cells in kidney in mice for SPECT imaging 33. By immunohistochemistry examination on the excised tissues, it was concluded that the injected radiolabelled cells remained viable at 24 h after transplantation. Chin *et al*. labelled isolated swine mensenchymal stem cells with [^111^In]oxine and yielded 42 MBq/million cells 34. The cell viability was tested up to 48 h after labelling and remained 95 % at all times. In the study reported by Brenner *et al*., one million hematopoietic progenitor cells were radiolabelled with 30 MBq [^111^In]oxine and the cell viability was impaired by 30 % after 48 h incubation but was not affected after 24 h incubation 35. Considering the relative low radioactivity doses used in this study (i.e. 1 MBq/5 million cells and 10-15 MBq/5 million cells for gamma counting and SPECT imaging, respectively) and the studied time points (up to 24 h), it can be anticipated that most of the cells were still viable before injecting to animals.

From SPECT/CT images, it can be observed that majority of the radiolabelled γδ T cells accumulated in the lungs within 30 min post injection, before being redistributed mainly to the liver and spleen over time **(Figure 2)**. In contrast, [^111^In]tropolone alone showed prolonged blood circulation and was measured 40.6±2.9 % ID in blood at 4 h post-injection by γ-counting and remained 16.3±1.8 at 24 h (data not shown. The unique kinetic biodistribution pattern of adoptively transferred γδ T cells and low levels of γδ T cells seen in the peripheral blood after administration, even at early time-points, have been reported in clinical 14 and pre-clinical studies 24. Interestingly, a similar pattern of biodistribution of other types of human T cells, for example chimeric antigen receptor (CAR) T cells, have been reported in murine models 22, correlating well with our SPECT/CT and gamma counting results. Quantitative *in vivo* tissue biodistribution of γδ T cells has been studied in syngeneic 4T1 breast cancer model and in xenogeneic MDA-MB-231 breast cancer model implanted in the mammary fat pad, using [^111^In]oxine 24 and [^89^Zr]oxine radiolabelling 23, respectively. In the former study, the uptake of the indigenous mouse [^111^In]γδ T cells in the 4T1 tumours was quantified as 2.6 %ID/g tumour, 48 h post injection. In the latter study, tumour uptake of [^89^Zr]human γδ T cells was measured as 1.2 %ID/g tumour 48 h post injection. In our study, IP tumours had a similar accumulation of γδ T cells to SC tumours of ~1.5 %ID/g at 24 h post injection. The differences in tumour type (breast tumours vs melanoma tumours), species origin of the γδ T-cells or the examination time after T cell transfer render direct comparison difficult.

This work represents a pioneering comparative study of the accumulation of γδ T cells among tumours in different locations. We hypothesise that there is a link between level of γδ T cell localisation to tumours and efficacy of the therapy. In many cases, tumours have metastasised and reside in different locations. By studying uptake of γδ T cell in tumours in different locations, we hope to gain further insight into what types of tumours may benefit from this treatment. Several papers have undertaken immunohistochemical staining of human γδ T cells in tumour-bearing mice in order to look at the effect of pre-injection of N-BPs on tumour accumulation of γδ T cells. It has been shown that infiltration of γδ T cells was significantly (p < 0.05) higher in adrenal gland tumours of mice receiving both γδ T cells and free ZOL than when injected with γδ T cells alone 7. A similar result was found in another study, which used γδ T cells in combination with the N-BP pamidronate (PAM). Immunohistochemical analysis of SC prostate tumours showed the presence of γδ T cells only when mice had been treated with both PAM and γδ T cells 10. Our results showed no significant difference in the tumour levels of γδ T cells between mice pre-treated with free or liposomal ALD, compared to PBS. This is in contrast with a recent study by Man *et al*. which found using gamma counting, a technique used in our study, pre-injection of liposomal alendronate 4 day previously enhanced tumour uptake of [^89^Zr]γδ T cells from 1.2 % to 2.1 %ID/g tumour 48 h post the adoptive cell transfer in a xenograft breast cancer model 23. In addition to difference in the tumour model between the two studies, in our study, the pre-injection of L-ALD and gamma counting were performed at 24 h prior and 24 h post the adoptive cell transfer, respectively. It is possible that it was too early to see the increase of γδ T cells in tumours using this dosing schedule.

One may suggest that since no difference in tumour levels of γδ T cells was achieved following L-ALD injection, this therapy may not be effective in sensitising γδ T cells. This is not the case as in our recently published work we have shown that human IFN-γ was only detected in the serum of the mice pre-treated with L-ALD prior to γδ T cells therapy 20. Additionally, only the combinatory treatment of L-ALD and γδ T cells led to a significant reduction in tumour growth in the experimental metastatic lung melanoma model. It is worth mentioning multiple injections of γδ T cells were performed in our 20, 21 and others' therapy studies 7, 10 (>3 times, weekly based) where an improvement in therapy was noticed.

The lungs in the SC model were shown to have γδ T cell uptake of 7.4 ± 3.0 % ID/g lung while tumour-bearing lungs had a significantly higher uptake of 22.6 ± 7.1 % ID/g lung (*p* = 0.03) (**Figure 4**, PBS group). Surprisingly but also interestingly, the IP model exhibited the highest γδ T cell uptake (37.1 ± 20.1 % ID/g lung) among the three models. Our findings indicate that physiological changes caused by the presence of metastatic lung, SC or IP tumours lead to a change in the biodistribution of γδ T cells. The pulmonary immune status in patients or preclinical animal models with peritoneal cancer was rarely discussed. Further studies are required to confirm the recruitment of γδ T cell by assessing the immune cell populations within the tumours and tumour draining lymph nodes by flow cytometry or immunohistochemistry.

L-ALD concentrations in lung tumour tissues were calculated by extrapolations from gamma counting studies. Assuming that ALD does not undergo immature release from the liposomes, it is expected to reach concentrations in the range of 4.2-16.15 nmol ALD/g in lung tissues. These concentrations, despite being 10-fold lower than needed for *in vitro* studies, seem to be sufficient to result in tumour growth delay *in vivo* 20, 21. This suggests that L-ALD is not homogenously distributed throughout the lung tissues, probably with higher concentrations in tumour tissues. In the IP model, variable uptake of L-ALD in tumour nodules was observed (**Figure 1D**) which is expected as tumour cells spread throughout the peritoneal cavity. Since the tumour uptake is a result of passive targeting based on the enhanced permeability and retention effect, vascularisation status will be varied between small and large tumour nodules within the peritoneum thus affecting the uptake efficiency. In fact, the first liposome-based nanomedicine, Doxil^®^, was approved to treat advanced ovarian cancer 36 which is often well vascularised 37. The relatively high uptake of L-ALD in IP tumour nodules indicates the therapeutic potential and this feature can be utilised in future studies in combination with γδ T cell therapy and/or incorporating with tumour targeting ligands 38 in the liposomal formulation to treat ovarian cancer or peritoneal metastasis.

## Conclusion

*In vivo* biodistribution studies of [^111^In]γδ T cells in mice agreed with other reports describing T-cell organ biodistribution with highest accumulation in the liver and spleen. Despite the comparable L-ALD uptake in all tumour types, γδ T cells showed highest uptake in the melanoma tumour-bearing lung model and lungs of mice implanted in the IP cavity. Lower γδ T cell count was found in the SC and IP tumours. Dosimetry studies focusing on correlating γδ T cells levels in *in vivo* tumours and therapeutic responses may help us understand better the impressively fast-growing field of T-cell based immunotherapy. Conclusions from the current study can only apply to the melanoma tumour model in NSG mice so further tests need to be carried out on other types of mice tumours.

## Figures and Tables

**Table 1:** Characterisation of liposome formulations

	Size (nm) ^[b], [e]^	Polydispersity	Zeta potential (mV) ^[a], [e]^	%EE^[c], [e]^	Radiolabelling efficiency^[d], [e]^
**L-ALD**	156.6 ± 0.9	0.051 ± 0.02	-12.5 ± 0.6	6.2 ± 0.2	-
**DTPA-L-ALD**	152.4 ± 2.9	0.058 ± 0.013	-11.1 ± 0.8	5.9 ± 0.6	82.6 ± 4.2

[a] Hydrodynamic diameter measured with dynamic light scattering. [b] Analysed with electrophoretic light scattering using 10 mM NaCl. [c] Measured with copper sulphate UV spectroscopy method. [d] Measured with quantitative autoradiography counting. [e] Data are represented as mean ± SD

## References

[1] D'Asaro M, La Mendola C, Di Liberto D (2010). V gamma 9V delta 2 T lymphocytes efficiently recognize and kill zoledronate-sensitized, imatinib-sensitive, and imatinib-resistant chronic myelogenous leukemia cells. J Immunol.

[2] Bernhard H, Neudorfer J, Gebhard K (2008). Adoptive transfer of autologous, HER2-specific, cytotoxic T lymphocytes for the treatment of HER2-overexpressing breast cancer. Cancer Immunol Immunother.

[3] Pockaj BA, Sherry RM, Wei JP (1994). Localization of 111indium-labeled tumor infiltrating lymphocytes to tumor in patients receiving adoptive immunotherapy. Augmentation with cyclophosphamide and correlation with response. Cancer.

[4] Slaney CY, Kershaw MH, Darcy PK (2014). Trafficking of T cells into tumors. Cancer Res.

[5] Benzaid I, Monkkonen H, Bonnelye E (2012). In vivo phosphoantigen levels in bisphosphonate-treated human breast tumors trigger Vγ9Vδ2 T-cell antitumor cytotoxicity through ICAM-1 engagement. Clin Cancer Res.

[6] Benzaid I, Monkkonen H, Stresing V (2011). High phosphoantigen levels in bisphosphonate-treated human breast tumors promote Vgamma9Vdelta2 T-cell chemotaxis and cytotoxicity in vivo. Cancer Res.

[7] Di Carlo E, Bocca P, Emionite L (2013). Mechanisms of the antitumor activity of human Vgamma9Vdelta2 T cells in combination with zoledronic acid in a preclinical model of neuroblastoma. Mol Ther.

[8] Kabelitz D, Wesch D, Pitters E (2004). Characterization of tumor reactivity of human V gamma 9V delta 2 gamma delta T cells in vitro and in SCID mice in vivo. J Immunol.

[9] Parente-Pereira AC, Shmeeda H, Whilding LM (2014). Adoptive immunotherapy of epithelial ovarian cancer with Vgamma9Vdelta2 T cells, potentiated by liposomal alendronic acid. J Immunol.

[10] Santolaria T, Robard M, Leger A (2013). Repeated systemic administrations of both aminobisphosphonates and human Vgamma9Vdelta2 T cells efficiently control tumor development in vivo. J Immunol.

[11] Sato K, Kimura S, Segawa H (2005). Cytotoxic effects of gammadelta T cells expanded ex vivo by a third generation bisphosphonate for cancer immunotherapy. Int J Cancer.

[12] Yuasa T, Sato K, Ashihara E (2009). Intravesical administration of gammadelta T cells successfully prevents the growth of bladder cancer in the murine model. Cancer Immunol Immunother.

[13] Kobayashi H, Tanaka Y, Yagi J (2007). Safety profile and anti-tumor effects of adoptive immunotherapy using gamma-delta T cells against advanced renal cell carcinoma: a pilot study. Cancer Immunol Immunother.

[14] Nicol AJ, Tokuyama H, Mattarollo SR (2011). Clinical evaluation of autologous gamma delta T cell-based immunotherapy for metastatic solid tumours. Br J Cancer.

[15] Wada I, Matsushita H, Noji S (2014). Intraperitoneal injection of in vitro expanded Vgamma9Vdelta2 T cells together with zoledronate for the treatment of malignant ascites due to gastric cancer. Cancer Med.

[16] Feurle J, Espinosa E, Eckstein S (2002). Escherichia coli produces phosphoantigens activating human gamma delta T cells. J Bio Chem.

[17] Weiss HM, Pfaar U, Schweitzer A (2008). Biodistribution and plasma protein binding of zoledronic acid. Drug Metab Dispos.

[18] Shmeeda H, Amitay Y, Tzemach D (2013). Liposome encapsulation of zoledronic acid results in major changes in tissue distribution and increase in toxicity. J Control Release.

[19] Epstein H, Gutman D, Cohen-Sela E (2008). Preparation of alendronate liposomes for enhanced stability and bioactivity: in vitro and in vivo characterization. AAPS J.

[20] Hodgins NO, Al-Jamal WT, Wang JT (2016). In vitro potency, in vitro and in vivo efficacy of liposomal alendronate in combination with gammadelta T cell immunotherapy in mice. J Control Release.

[21] Hodgins NO, Al-Jamal WT, Wang JTW (2017). Investigating in vitro and in vivo αvβ6 integrin receptor-targeting liposomal alendronate for combinatory γδ T cell immunotherapy. J Control Release.

[22] Parente-Pereira AC, Burnet J, Ellison D (2011). Trafficking of CAR-engineered human T cells following regional or systemic adoptive transfer in SCID beige mice. J Clin Immunol.

[23] Man F, Lim L, Volpe A (2018). In Vivo PET Tracking of 89Zr-Labeled Vγ9Vδ2 T Cells to Mouse Xenograft Breast Tumors Activated with Liposomal Alendronate.

[24] Beck BH, Kim HG, Kim H (2010). Adoptively transferred ex vivo expanded gammadelta-T cells mediate in vivo antitumor activity in preclinical mouse models of breast cancer. Breast Cancer Res Treat.

[25] New RRC (1990). Liposomes: a practical approach. Oxford; New York; New York: IRL Press; Oxford University Press.

[26] Koba M, Koba K, Przyborowski L (2008). Application of UV-derivative spectrophotometry for determination of some bisphosphonates drugs in pharmaceutical formulations. Acta Pol Pharm.

[27] DiCara D, Rapisarda C, Sutcliffe JL (2007). Structure-function analysis of Arg-Gly-Asp helix motifs in alpha v beta 6 integrin ligands. J Biol Chem.

[28] Kotze HF, Heyns AD, Lotter MG (1991). Comparison of oxine and tropolone methods for labeling human platelets with indium-111. J Nucl Med.

[29] Datz FL (1994). Indium-111-labeled leukocytes for the detection of infection: current status. Semin Nucl Med.

[30] Segall GM, McDougall IR (1986). Diagnostic value of lung uptake of indium-111 oxine-labeled white blood cells. AJR Am J Roentgenol.

[31] Millet I, Bouic-Pages E, Hoa D (2011). Growth of breast cancer recurrences assessed by consecutive MRI. BMC Cancer.

[32] Jin Y, Kong H, Stodilka RZ (2005). Determining the minimum number of detectable cardiac-transplanted 111In-tropolone-labelled bone-marrow-derived mesenchymal stem cells by SPECT. Phys Med Biol.

[33] Tai JH, Nguyen B, Wells RG (2008). Imaging of Gene Expression in Live Pancreatic Islet Cell Lines Using Dual-Isotope SPECT. J Nucl Med.

[34] Chin BB, Nakamoto Y, Bulte JW (2003). 111In oxine labelled mesenchymal stem cell SPECT after intravenous administration in myocardial infarction. Nucl Med Commun.

[35] Brenner W, Aicher A, Eckey T (2004). 111In-Labeled CD34+ Hematopoietic Progenitor Cells in a Rat Myocardial Infarction Model. J Nucl Med.

[36] Green AE, Rose PG (2006). Pegylated liposomal doxorubicin in ovarian cancer. Int J Nanomedicine.

[37] Steinkamp M, Kanigel-Winner K, Davies S (2013). Ovarian Tumor Attachment, Invasion, and Vascularization Reflect Unique Microenvironments in the Peritoneum: Insights from Xenograft and Mathematical Models.

[38] Shmeeda H, Tzemach D, Mak L (2009). Her2-targeted pegylated liposomal doxorubicin: Retention of target-specific binding and cytotoxicity after in vivo passage. J Control Release.

