# Supplementary material for: Organ Biodistribution of Radiolabelled γδ T Cells Following Liposomal Alendronate Administration in Different Mouse Tumour Models
Source: Nanotheranostics. 2020 Feb 6;4(2):71–82. doi: 10.7150/ntno.32876 (PMC7064741; doi:10.7150/ntno.32876)
Supplement: Supplementary file 1 — Supplementary figures. [file ntnov04p0071s1.pdf]

Organ Biodistribution of Radiolabelled δγ T Cells Following Liposomal Alendronate Administration in Different Mice Tumour Models

Julie T-W. Wang[†,], Naomi O. Hodgins[†], Wafa' T. Al-Jamal, John Maher, Jane K. Sosabowski and Khuloud T. Al-Jamal**

Dr. J. Wang, Dr. N. Hodgins and Professor K.T. Al-Jamal
150 Stamford Street,
Institute of Pharmaceutical Science
King's College London
Franklin-Wilkins Building
150 Stamford Street
London SE1 9NH, UK
E-mail: khuloud.al-jamal@kcl.ac.uk

Dr. W.T. Al-Jamal
School of Pharmacy
Queen's University Belfast
Whitla Medical Building Block 97B MBC Site
Belfast, United Kingdom

Dr. Jane Sosabowski
Barts Cancer Institute
Queen Mary University of London
London EC1M 6BQ, UK

Dr. J. Maher
King's College London
Division of Cancer Studies
Guy's Hospital
London SE1 9RT, UK

* Corresponding author - E-mail: khuloud.al-jamal@kcl.ac.uk; julie.tzu-wen.wang@kcl.ac.uk

SUPPLEMENTARY FIGURES

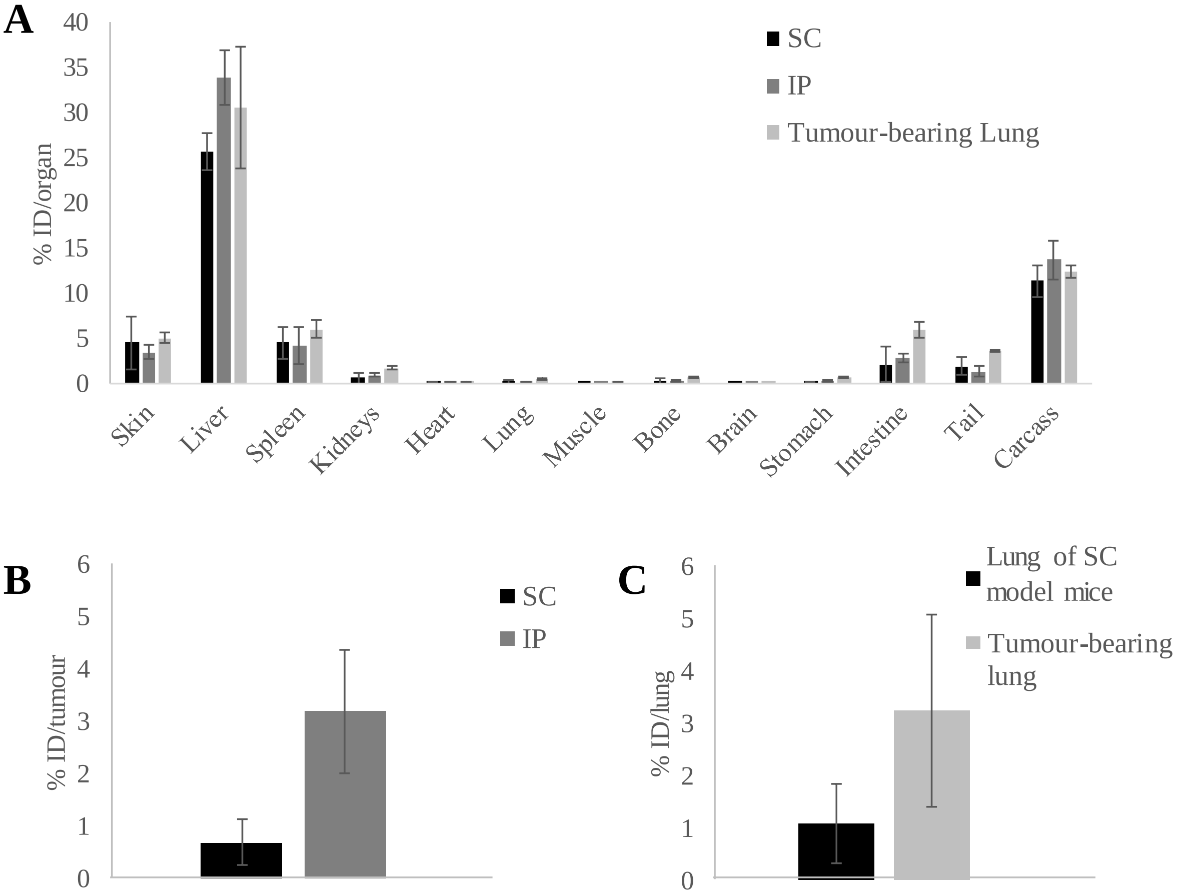

Figure S1*: In vivo* Biodistribution of radiolabelled [^{111}In]L-ALD in A375Pβ6 tumours, after single dose administration *via* tail vein injection in NSG mice. NSG mice were inoculated with the A375Pβ6 cell line to form subcutaneous (SC), intraperitoneal (IP) or pseudo-metastatic lung tumours. Mice were i.v. injected with [^{111}In]L-ALD at a dose of 2 μmol lipid/mouse. After 24 h the mice were sacrificed and the amount of liposomes was quantified by gamma counting. **(A)** Organ biodistribution of [^{111}In]L-ALD expressed as per cent injection dose organ (%ID). **(B)** SC-tumour and IP-tumours uptake of [^{111}In]L-ALD expressed as %ID. **(C)** Tumour-bearing lung and healthy lung uptake of [^{111}In]L-ALD expressed as %ID. Data was expressed as mean ± SD (n=4)

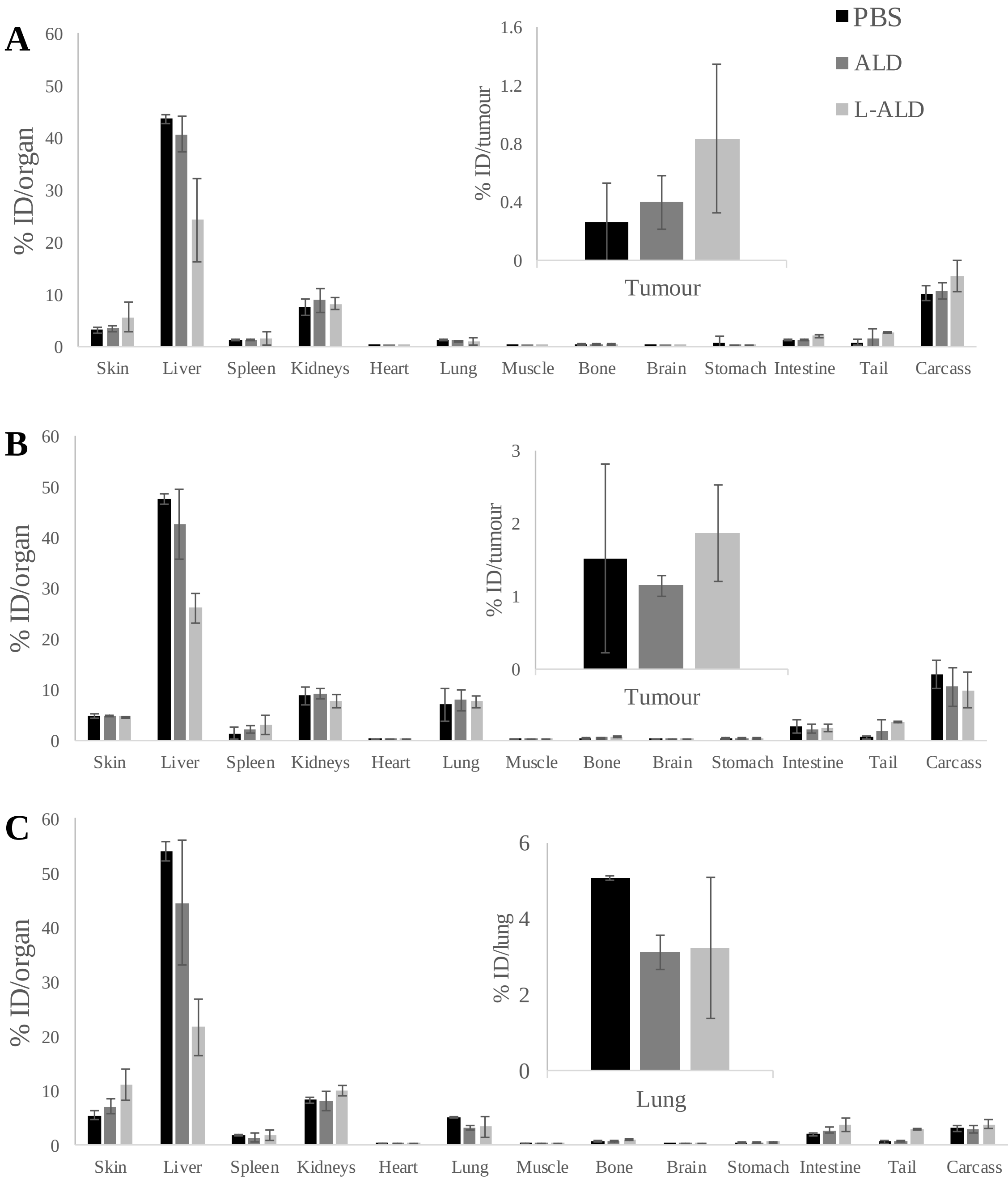

Figure S2: *In vivo* biodistribution of radiolabelled γδ T cells in A375Pβ6 tumour bearing NSG mice, after single dose administration *via* tail vein injection. NSG mice were inoculated with luciferase-expressing A375Pβ6 cell line to form **(A)** subcutaneous (SC), **(B)** intraperitoneal (IP) or **(C)** pseudo-metastatic lung tumours. Mice were i.v. injected with $[^{111}In]$γδ T cells at a dose of 5 x 10^6 γδ T cells/mouse. Mice were pre-treated with 0.5 μmol ALD or L-ALD, 24 h prior to injection of γδ T cells. After 24 h the mice were sacrificed and the amount of γδ T cells was quantified by gamma counting. Results are expressed as percentage injection dose (%ID) per organ. Data was expressed as mean ± SD (n=4).
